# Animal models of rheumatoid arthritis‐associated interstitial lung disease

**DOI:** 10.1002/iid3.377

**Published:** 2020-11-20

**Authors:** Li Xiong, Liang Xiong, Hong Ye, Wan‐Li Ma

**Affiliations:** ^1^ Department of Respiratory and Critical Care Medicine, Union Hospital, Tongji Medical College Huazhong University of Science and Technology Wuhan China; ^2^ Department of Pathophysiology, School of Basic Medicine, Tongji Medical College Huazhong University of Science and Technology Wuhan China; ^3^ Key Laboratory of Respiratory Diseases Ministry of Health of China Wuhan China

**Keywords:** animal models, autoimmune disease, interstitial lung disease, lung inflammation, rheumatoid arthritis

## Abstract

**Background:**

Rheumatoid arthritis‐associated interstitial lung disease (RA‐ILD) is an irreversible pathologic condition of unknown cause, commonly involving the joint and the lung with variable amounts of fibrotic change. In contrast to rheumatoid arthritis or other chronic interstitial lung diseases such as interstitial pulmonary fibrosis, there is so far no extensively accepted or implemented animal model for this disease.

**Aims:**

To provide guidance for those who are investigating the pathogenesis of RA‐ILD with animal models.

**Materials and Methods:**

An analysis of papers from PubMed during 1978‐2020.

**Results:**

We outline the present status quo for animal models of RA‐ILD about their modeling methods and pathogenesis, compare their pros and cons with respect to their ability to mimic the clinical and histological features of human disease and discuss their applicability for future research.

**Discussion:**

There is no doubt that these animal models do provide valuable information relating to the pathogenesis of RA‐ILD and the development of effective therapeutic drugs. Nevertheless, these animal models can not entirely recapitulate clinical pathology and have some limitations in experimental research application. Therefore, it should be emphasized that we should improve and explore animal models in more accordance with the pathogenesis and clinical characteristics of human RA‐ILD.

**Conclusion:**

These established animal models of the disease can significantly progress our understanding of the etiology of RA‐ILD, the fundamental mechanisms of its pathogenesis and the identification of new bio‐markers, and can contribute to the development and implementation of novel treatment strategies.

## Graphical abstract

The manuscript summarizes the characteristics of animal models about rheumatoid arthritis‐associated interstitial lung disease (RA‐ILD), which can provide reference for those researchers who are interested in investigating the pathogenesis of RA‐ILD.

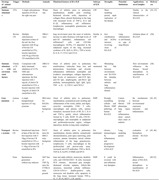

## 1 INTRODUCTION

Rheumatoid arthritis‐associated interstitial lung disease (RA‐ILD) is a systematic autoimmune disease characterized by joint lesions^1^ and diffuse interstitial pulmonary changes,^2^ accompanying poor prognosis.^3^, ^4^, ^5^ The possibility of developing RA‐ILD increases over time after diagnosis of rheumatoid arthritis (RA) and the median survival of RA‐ILD is about 2.6–3.5 years.^4^, ^6^ Usual interstitial pneumonitis (UIP) and nonspecific interstitial pneumonitis (NSIP), these two subtypes predominate in RA‐ILD.^7^, ^8^, ^9^ It includes other relatively rare subtypes: desquamative interstitial pneumonitis (DIP), respiratory bronchiolitis‐associated interstitial lung disease (RB‐ILD), organizing pneumonia (OP), lymphocytic interstitial pneumonitis, pleuroparenchymal fibroelastosis, and diffuse alveolar damage, according to its histological features.^7^, ^10^ The UIP pattern featuring heterogeneity tends to display fibrotic disease process with subpleural and paraseptal matrix deposition,^11^, ^12^, ^13^, ^14^ while the NSIP pattern featuring homogeneity trends to appear inflammatory infiltration in nature with alveolar wall thickening.^12^, ^13^, ^15^, ^16^

In patients of RA‐ILD, RA usually develops before onset of ILD.^9^ Yet, ILD precedes onset of RA^17^, ^18^, ^19^, ^20^ and both conditions occur simultaneously in some patients of RA‐ILD.^9^ The pathogenesis of RA‐ILD is still unclear and there is a lack of effective treatment. Therefore, it is of great importance to establish appropriate experimental animal models of RA‐ILD.

At present, common methods to induce RA‐ILD include adjuvant, Collagen Type II (CII), combination of organic dust extract (ODE) and CII, zymosan, aberrant expression of specific genes through transgenic systems or gene mutation. In this review, we have a relatively comprehensive introduction for present animal models of RA‐ILD to provide reference for the basic research, drug screening and efficacy evaluation of RA‐ILD.

## 2 ANIMAL MODELS OF RA‐ILD

### 2.1 Animal model of RA‐ILD induced by immune stimulants

#### 2.1.1 Complete Freund's adjuvant

Freund's adjuvant is the common adjuvant, which is widely used to induce onset arthritis in animal models. It includes Freund's complete adjuvant (FCA) and Freund's incomplete adjuvant (FIA). The difference between FCA and FIA is that FCA contains *Mycobacterium tuberculosis* in the form of inactivated or dead BCG vaccine.^21^ It also means that FCA is comprised of FIA and *M. tuberculosis*.

Adult Wistar rats were given a single injection of 0.1 ml FCA into the right rear paw.^22^, ^23^ After the injection of adjuvant, the first joint swelling appeared from 12 h to Day 3. Likely, body weight increased until Day 17 and then decreased until the termination of experiment. The secondary joint swelling occurred on Day 18 until the end Day of 28. After 20 days, the FCA‐treated rats began to manifest erythema and paw swelling at the contralateral joint. The lung of FCA‐treated rats developed the thickened alveolar walls and inflammatory cell infiltration comprised by lymphocytes, eosinophilic granulocytes, and macrophages on Day 21. Furthermore, the rats displayed apparent deposition of collagen fibers and pleural thickening in the lung on Day 28 with increased serum and pulmonary levels of pro‐inflammatory cytokines such as tumor necrosis factor‐α (TNF‐α), interleukin‐6 (IL‐6), and IL‐1β, in addition to high pulmonary transforming growth factor (TGF)‐β1 expression.^22^, ^23^

The histopathological pattern of this animal model is similar to the clinical characteristics of RA‐ILD. The mechanism in this animal model is that FCA may elicit inflammation in joints and lung due to macrophage phagocytosis and circulation.^21^ Merit is that this model is characterized by easy operation, short modeling time and low experiment cost. In addition, inflammation and fibrosis were mainly distributed in the pleural and subpleural area, which was similar to the pathological distribution of clinical RA‐ILD especially RA associated with UIP. The demerit is that this animal model of the disease has a certain self‐limitation.^24^

#### 2.1.2 Bovine Collagen Type II

CII is extensively used to induce onset of arthritis in RA animal models.^25^, ^26^ Presumably, anti‐CII antibodies react with the CII that is a major protein in cartilage, causing joint inflammation and bone erosion. Several investigators have found that anti‐citrullinated protein antibodies (ACPAs), reacting with various citrullinated proteins, can not only be generated at joints, but also in the lung^27^ and ACPAs are closely related to RA‐ILD.^28^, ^29^ However, the relevant pulmonary manifestation after CII immunization has scarcely been reported. Therefore, the researcher conducted this experiment and found it suitable for bovine Collagen Type II (bCII) to induce onset of RA‐ILD in DBA/1 mice.

On Day 0, the DBA/1 mice were given an injection of 50 μg of bCII emulsified in CFA into the base of the tail.^30^ On Day 21,105 and 161, the mice were given booster injections of 50 μg of bCII emulsified in IFA. On Day 7, the CII‐treated mice began to develop inflammatory cell aggregates in the subpleural region accompanying increasing anti‐cyclic citrullinated peptide (CCP) antibodies associated with ACPAs. Anti‐CII antibodies began to develop and inflammatory arthritis occurred on Day 14. Thus, this indicated that lung inflammation preceded the onset of arthritis in CII‐treated mice. On Day 49, the mice developed an increase in ankle thickness. There were increased inflammatory cytokines of IL‐1β, IL‐6, IFN‐γ, TNF‐α levels, IL‐4, IL‐17, CCL3, and CCL4, and high levels of CD4+ T cells CD8+ T cells, Th1 cells, Th2 cells, Th17 cells, CD4+CD25+Foxp3+ regulatory T cells, and macrophages in the lungs of CIA mice. Of note, CD11bhi macrophages, regarded as interstitial macrophages,^31^, ^32^, ^33^ dominated the inflammatory cellular infiltration. In addition, ACPAs and Complement 3 (C3) were deposited in the subpleural region. However, on Day 182 or 189, lung inflammation could be reversed and there is no indication of pulmonary fibrosis.

The merit about this model is that it can be used for initiation phase of RA‐ILD to understand how citrullination and ACPAs interacts. In addition, it can reflect the inflammation phase of RA‐ILD due to inflammation area is mainly distributed in subpleural area, which is similar to the pathological distribution of preclinical RA‐associated UIP. Breach of tolerance and generation of autoantibodies toward self and collagen make the model the gold standard for studies.^34^ However, there is no fibrosis and inflammation is self‐limiting in the lung. Moreover, this model cannot replicate the characteristics of chronic and irreversible progress of human RA‐ILD.

#### 2.1.3 Chicken CII and ODE

Environmental factors play an important role in RA‐ILD development, especially in long‐term and repetitive inhalation relevant to occupational exposure of farmers and workers^35^ who were inevitably faced with OD. Notably, components of OD were trace metals, predominance of gram‐positive bacteria (98%), and high muramic acid,^36^, ^37^ which can cause macrophage dysfunction, airway, and lung parenchymal inflammation.^36^ Simultaneously, lots of research has shown that CII can induce onset of inflammatory arthritis in the murine. Considering these, combination of the ODE and chicken CII resulted in specific establishment of an animal model of RA‐ILD.

On Day 1, DBA/1J strain mice were given a subcutaneous injection of 2 mg/ml of chick CII emulsified in FCA.^38^ At Week 3, the mice were given the booster injection of chick CII emulsified in FIA. At the same time, the mice received daily treatment with intranasal inhalation of 12.5% ODE for 5 weeks continuously (weekends excluded).

Inflammatory arthritis began at Week 3 and became severely obvious at Week 5. CII + ODE‐treated mice displayed a decline of bone mineral density (BMD) in both the proximal tibia and calcaneus compared with CII‐treated mice. In addition, CII + ODE‐treated mice demonstrated a reduction of bone volume, trabecular thickness, and polar moment of inertia in the calcaneus compared with CII‐treated mice. In short, CIA + ODE (coexposure) treatment resulted in trabecular bone loss and deterioration. Moreover, the coexposure group developed more severe arthritis symptom than ODE‐treated group.

After 5 weeks, the lung in coexposure mice displayed increased lymphoid aggregates, bronchiolar, and alveolar inflammation comprised of lung neutrophils and exudative macrophages, with collagen deposition and increased hyaluronan, fibronectin accompanying high levels of pentraxin‐2, anti‐CCP IgG antibody, Type II collagen IgG, amphiregulin, anti‐MAA IgG antibody, immunoglobulin (IgG, IgM, and IgA), TNF‐α, IL‐6, and neutrophil chemoattractants (CXCL1 and CXCL2). Nevertheless, there was less inflammation infiltration in alveolar compartment and more extracellular matrix protein deposition in the CII + ODE group versus the ODE group. In summary, lung inflammation was shifted to pulmonary interstitial changes in the coexposure treated mice compared with ODE‐treated mice. Interestingly, the female mice manifested suppressed inflammatory cell and arthritis than male mice under the CII + ODE treatment.

The merit is that this novel animal model vividly mimics what influence the environmental factors have on RA‐ILD individuals and the interplay between arthritis and lung inflammatory disease that repetitive inhalant exposure to an environmental inflammatory agent rich in microbe aggravates articular inflammation, bone destruction, as well as cartilage erosion and conversely arthritis elicitation appears to impact the inflammatory/interstitial lung processes. CII and inhalation induced arthritis and fibrosis, which can simulate the pathological process of human RA‐ILD in the environment. This preclinical animal model could be further exploited to develop potential interventional strategies to mitigate arthritis and lung disease such as through respiratory protection and environmental improvement. Also, this animal model can be used to investigate biological sex differences in morbidity and mortality of human RA‐ILD. However, the operation on this established model is relatively complex, because it is necessary to ODE and have the mouse going through specific inhalation every day to better simulate certain circumstances where human are.

### 2.2 Animal model of RA‐ILD induced by genetic engineering

#### 2.2.1 Animal model of RA‐ILD by gene mutation

The SKG mice have a spontaneous point mutation in the second SH2 domain of ZAP70 relevant to tyrosine‐phosphorylated immunoreceptor tyrosine‐based activation motifs of TCR‐ζ and CD3 chains, as a consequence of decreased T‐cell receptor signaling in thymocytes, which impaired positive and negative CD4+ T cell selection in the thymus enabling autoreactive CD4+T cells to escape into the periphery.^39^, ^40^ Furthermore, it consequently displays the phenotype of immunological diseases and might be crucial to the development of RA‐ILD.

SKG mice failed to develop RA‐ILD under specific‐pathogen‐free (SPF) conditions despite the fact that thymic production of autoimmune CD4+T cells persisted in the periphery, but intraperitoneal injection of zymosan especially β‐glucans can cause the development of an RA‐ILD clinical phenotype in the same condition above mentioned.^41^ Zymosan came from crude yeast cell wall extract, the main constituents of which were β‐glucans.^41^ Interestingly, SKG mice spontaneously began to manifest chronic arthritis about 2 months of age and developed the RA‐ILD disease about 6 months of age in a conventional environment containing certain microbial compounds, but not under SPF conditions.^41^ This suggested that a specific combination of aberrant expression in thymus CD4+ T cell and exposure to particular microbes was of vital importance to the development of RA‐ILD, but either one was not.

SKG mice were given a single intraperitoneal injection of 5 mg zymosan to provoke severe arthritis and advance onset of ILD under SPF condition.^42^, ^43^, ^44^ The mice began to display symmetrical joint swelling and developed inflammatory arthritis of the wrists, ankles, and digits characterized by synovial inflammation with moderate cartilage damage and narrowing of the joint space at 2–3 weeks after zymosan injection. Symmetrical joint swelling began in small joints of the digits and progressed to larger joints, accompanying severe synovitis with massive subsynovial infiltration of neutrophils, lymphocytes (mainly CD4+ T cells), macrophages, and plasma cells. Likewise, it presented villus proliferation of synoviocytes with pannus formation and neovascularization, and neutrophil‐rich exudates in the joint cavity with progression of synoviocyte proliferation. It was predicted that this unique combination of high self‐reactivity of T cells and susceptibility of synovial cells to inflammatory stimuli leads to predominant development of erosive arthritis in SKG mice. Moreover, the severity of joints swelling gradually increased before peaking in severity at 6–10 weeks after zymosan injection and then declined but remained elevated in mice 16 weeks after zymosan injection. Destruction and fusion of the subchondral bones, joint dislocation, and osteoporosis appeared at 8–12 months of age. Also, SKG mice developed high titers of rheumatoid factor (RF) and other autoantibodies in the circulation with increased pro‐inflammatory cytokines of IL‐1, IL‐6, and TNF‐a as well as incremental chemical mediators that destroy the surrounding cartilage and bone.

At 12–16 weeks after zymosan injection, arthritic SKG mice developed a patchy subpleural and peribronchovascular mixed inflammation formed by CD4+ T cells, CD8+ T cells, B220+ B cells, CD11b+ macrophages, and neutrophils, with variable amounts of collagen but not large areas of excessive collagen deposition in the areas of accumulated cells. There was also a small increase in the number of lavaged cells: macrophages, neutrophils, and lymphocytes. The increased fibrosis seen in zymosan‐injected SKG mice was associated with active TGF‐β signaling in the lung parenchyma. Likewise, there was a decline in lung static compliance. The lung inflammation can involve up to 89% of the mice injected with zymosan. However, neither exposure to cigarette smoke nor bleomycin resulted in the onset of arthritis in SKG mice.^42^ Furthermore, anti‐cyclic citrullinated antibodies were present in the sera of arthritic SKG mice that were injected with zymosan, but not detected in the sera of the mice exposed to cigarette smoke.

SKG mice injected with zymosan closely mirroring human RA‐ILD have their own advantages and disadvantages. SKG mice can represent a useful model that will allow the investigation of the immune and inflammatory mechanisms contributing to the development of interstitial pneumonia in the setting of autoimmune arthritis. The pulmonary manifestation of SKG mice following intraperitoneal zymosan injection strongly resembles NSIP pathology to that seen in human RA‐ILD. However, SKG mice did not develop a fibrotic UIP phenotype while there was increased deposition of collagen in the lung after 18 weeks post zymosan injection,^42^ which is not consistent with the course of RA‐ILD. Just lung involvement is insufficient to induce onset of arthritis in SKG mice.^42^ Furthermore, while the SKG mice with identical genetic background undergo the same circumstances, they can show various disease phenotype of joint and lung.

### 2.3 Transgenic animal models of RA‐ILD

#### 2.3.1 The D1CC transgenic mouse

The human class II transactivator (CIITA) gene was linked to the rat CII promoter and enhancer in DBA/1 mice through plasmid construction, which can result in aberrant expression of major histocompatibility complex Class II (MHC Class II).^45^ These transgenic DBA/1 mice were called D1CC transgenic mice. The D1CC transgenic mice treated by small amounts of bCII in adjuvant can induce onset of RA‐ILD in the D1CC transgenic mice.

The D1CC mice were given the first intradermal injection of 10 µg of bCII emulsified in an equal volume of CFA into the base of the tail on Day 0.^45^, ^46^ Mice received booster injections of bCII in the same location using IFA on Days 21, 42, and 63.^45^, ^46^ The erosive destruction of joints started in the early stages of inflammation for 2 months after the second booster injection. Eighty‐nine percent of D1CC mice developed articular symptom of joint swelling, redness, and heat after immunization.^45^ The mice displayed infiltration of inflammatory cells, proliferation of synoviocytes with pannus formation characterized by accumulation of an abundance of granulocytes, and erosion of bone. At the terminal stage of chronic inflammatory arthritis, it revealed the decline of bone mineral density and joint space narrowing and erosions at the patella, distal femur, proximal tibia as well as fibula, and severe ankylosis at the proximal interphalangeal and metacarpophalangeal joints, but without the appearance of osteophytes. Fifteen months after the second boost, the mice manifested the complete destruction of all joints and shortening of phalanges together with the high levels of anti‐CCP antibodies.

The infiltrating inflammatory cells in the lung damage were granulocytes, lymphocytes, T cells and macrophages with pneumocyte hyperplasia and fibrotic changes. Mixed cellular inflammation was distributed in the peribronchial and perivascular areas with fibrosis, deposition of newly synthesized elastic fibers, and increased anti‐cyclic citrullinated peptide antibodies for 6 months after the second boost. Serum SP‐D levels were significantly increased approximately 10 months after the first injection of bCII, which can be a suitable marker of lung damage in RA‐ILD. TNF‐α expression in macrophages, BAX expression in reactive pneumocytes, TGF‐β expression in fibroblastic cells, IL‐6 expression in plasmacytoid cells, and soluble collagen expression was high in the lung lesions in DICC mice 10 months following the first bCII injection.^46^ The number of 8‐OHdG‐positive epithelial and inflammatory cells was significantly increased in DICC mice at 10 months after bCII injection. These molecules play an important role in the pathophysiology of RA and fibrosis in humans during inflammation, apoptosis, and extracellular matrix production.^46^

Merit to this model is that it can be used in evaluation of biomarkers and general pathophysiology of chronic disease process in resembling human RA‐ILD, which can well mimic the chronic and progressive course of RA‐ILD. Demerit to this model is that this model needs long time to get molded and could not completely replicate the relevant pulmonary pathological manifestations of UIP the type of which is predominant in the RA‐ILD.

#### 2.3.2 The 3647 line of the tumor necrosis factor transgenic (TNF‐Tg) mouse

The 3647 line of TNF‐Tg (Tg3647)mouse strains carrying a single copy of human TNF transgene with a modified 3ʹ‐untranslated region (3ʹ‐UTR) exchanging for the 3ʹ‐modified β‐globin UTR,^47^, ^48^ began to appear the swollen ankle joints around 2 months of age and developed inflammatory erosive arthritis at the age of 3 months without any stimuli. The TNF‐Tg mouse strains, overexpressing the human TNF‐α gene, are usually evaluated at cross‐sectional time points of 3, 4, 5.5, and 12 months.^49^, ^50^, ^51^, ^52^

The Tg3647 mice developed knee and ankle arthritis with dysfunction of the joint draining lymph nodes featuring a progressive increase in their popliteal lymph node volumes in which activated monocytes, conventional dendritic cells, and CD21hi/CD23+ B cells accumulated, due to the increase in lymphatic trafficking from the synovial space. Furthermore, grip strength decreased as well. Grip strength and the total volume of the lymph node reflected disease progression and activity in inflammatory arthritis.

The most notable patterns of lungs in Tg3647 mice included interstitial cellular infiltrates, thickened alveolar septums, perivascular, and peribronchiolar inflammatory infiltrates as well as the formation of follicle‐like structures lacking a germinal center with no apparent interstitial fibrosis. There was a significant increase of CD11b+/CD11c+ double‐positive cells and apoptosis in the lung tissue in the Tg3647 mouse line, concomitant with other increased populations of monocytes and dendritic cells. In addition to this, there was also a significant increase in the serum levels of human TNF‐α, IL‐17, interferon‐γ–inducible protein 10, monocyte chemoattractant protein 1, leukemia inhibitory factor, and keratinocyte chemoattractant.

The merit to Tg3647 murine model is that it mimics a cellular NSIP pattern dominated by an interstitial accumulation of inflammatory cells with a small amount of fibrotic change. A prior inflammatory state of this animal model could be used to investigate into inflammation phase of RA‐ILD. In addition, it could be used to assess anti‐TNF therapies but can also be used for testing other biologics and small molecules could be reversed with anti‐inflammatory therapies.^48^ Moreover, the female Tg3647 mice manifested the earlier symptoms of arthritis, and developed the more severe pathology of interstitial lung disease, which resulted in shorter lifespans and more mortality than their male counterparts.^52^ It could be used as a suitable model to better understand the mechanisms underlying sexual dimorphism. However, the demerit to this model is that it is relatively unitary in nature for the reason that the process of human RA‐ILD is due to the interaction of multiple genes and multiple factors.^53^ In addition, this model takes a long time to establish and the experiment cost of it is high.

## 3 CONCLUDING REMARKS

So far, we have focused our review on the pathophysiological and histomorphological characteristics of preclinical disease models, in addition to their practical aspects including reproducibility and feasibility simulating human pathology in RA‐ILD (summarized in Table 1). There is no doubt that they do provide valuable information relating to the pathogenesis of RA‐ILD and the development of effective therapeutic drugs. Nevertheless, these animal models can not entirely recapitulate clinical pathology and have some limitations in experimental research application. Therefore, it should be emphasized that we should improve and explore animal models in more accordance with the pathogenesis and clinical characteristics of human RA‐ILD.

**Table 1:** Animal models of RA‐ILD: Pathogenic triggers, features of human disease, subtypes, strengths, and limitations

**Strategy of animal model**	**Trigger**	**Methods**	**Animals**	**Mimicked features of RA‐ILD**	**Subtype of ILD**	**Strengths**	**Potential limitations**	**Applications or novel insights**	**References**
Immune stimulants	CFA	A single subcutaneous injection of CFA at the right rear paw	Wistar rats	Onset of arthritis before pulmonary manifestation; double joint swelling; thickened alveolar walls, deposition of collagen fibers, pleural thickening in the lung with increased levels of TNF‐α, IL‐6, and IL‐1β, inflammatory cell infiltration: lymphocytes, eosinophilic granulocytes, and macrophages	UIP	Short modeling period, rapid replication of the disease		Similar to the clinical characteristics of RA‐ILD	Song et al.^22^ and Yang et al.^23^
	Bovine CII	Multiple subcutaneous Injections at base of the tail: the first injection with 50 μg of bovine CII emulsified in CFA; booster injections with 50 μg of bovine CII emulsified in IFA	DBA/1 mice	Lung involvement prior the onset of arthritis; increase in ankle thickness with high levels of anti‐CII antibodies; inflammatory cell infiltration(T cells and CD11bhi macrophages), ACPAs, C3 deposited in the subpleural region of the lung, increased cytokines of IL‐1β, IL‐6, IFN‐γ, TNF‐α levels, IL‐4, IL‐17, CCL3, and CCL4	Similar to UIP	How citrullination and ACPAs interacts involving onset of RA‐ILD	Lung inflammation is self‐limited, no sign of lung fibrosis	Initiation phase of RA‐ILD	Sato et al.^30^
Immune stimulants with environmental factors	Chicken CII and ODE	Coexposure with daily intranasal inhalation of 12.5% ODE and subcutaneous injections: the first injection with 2 mg/ml of chick CII emulsified in CFA; a booster injection with 2 mg/ml of chick CII emulsified in IFA	DBA/1J mice	Onset of arthritis before pulmonary manifestation; trabecular bone loss and deterioration; bronchiolar and alveolar inflammation infiltration: neutrophils and exudative macrophages; collagen deposition, high levels of pentraxin‐2, anti‐CCP IgG, anti‐CII IgG, amphiregulin, anti‐MAA IgG antibody, immunoglobulin (IgG, IgM, IgA), TNF‐α, IL‐6, CXCL1, and CXCL2	Not mentioned	Mimicking the link between environment and RA‐ILD; the interplay between arthritis and lung inflammatory disease		How environment influences the disease. Sexual dimorphism in manifestation of RA‐ILD	Poole et al.^38^
Gene mutation	Zymosan	A single intraperitoneal injection of 5 mg zymosan	SKG mice for BALB/c mice background	Onset of arthritis before pulmonary manifestation; symmetrical joint swelling and inflammation of the wrists, ankles, and digits, synovitis: neutrophils, CD4+ T cells, macrophages and plasma cells, pannus formation; increased levels of RF, IL‐1, IL‐6, and TNF‐a, patchy mixed inflammation formed by T cells, B220+ B cells, CD11b+ macrophages, and neutrophils in subpleural and peribronchovascular region, collagen deposition, and decline in lung static compliance	NSIP	Strongly resembling cellular and fibrotic NSIP pathology of human RA‐ILD	Cannot develop a fibrotic UIP phenotype, lung injury and inflammation alone cannot elicit onset of arthritis	the mechanisms between environmental exposures, immunity and inflammation	Keith et al.^42^ and Redente et al.^43^
Transgenic systems	Bovine CII	Intradermal injections at base of the tail: the first injection with 5–10 μg of bovine CII emulsified in CFA; a booster injection with 5–10 μg of bovine CII emulsified in IFA	D1CC mice for DBA/1 mice background	Onset of arthritis before pulmonary manifestation; chronic arthritis: uxtaarticular demineralization, joint space narrowing, joint erosions, pannus formation; mixed cellular inflammation of the lung: granulocytes, lymphocytes, T cells, macrophages in the peribronchial and perivas‐cular areas, increased anti‐CCP antibodies, TNF‐α, BAX, TGF‐β, IL‐6, LPO, 8‐OHdG, and SP‐D.	Not mentioned	Chronic, immunologically inflammatory and progressive development of RA‐ILD	Long modeling period	evaluation of biomarkers, chronic RA‐ILD model	Kanazawa et al.^45^ and Terasaki et al.^46^
	None	Spontaneous generation without any inducers	3647 line of TNF‐Tg mice for C57BL/6 mice ground	Knee and ankle arthritis: monocytes, dendritic cells, and CD21hi/CD23+ B cells; increased PLN volumes, decreased grip strength; thickened alveolar septums, follicle‐like structures, interstitial cellular infiltrates: CD11b+/CD11c+ double‐positive cells, monocytes and dendritic cells; apoptosis in the lung tissue, increased human TNF‐α, IL‐17, IP‐10, MCP‐1, LIF, and KC	NSIP	It could be reversed with anti‐inflammatory therapies		Inflammation phase of RA‐ILD, sexual dimorphism in manifestation of RA‐ILD	Wu et al.^49^, Fehrenbach et al.^50^, Wu et al.^51^, and Bell et al.^52^

Abbreviations: CFA,complete Freund's adjuvant; CII, collagen type II; IFA, incomplete Freund's adjuvant; IP‐10, interferon‐γ–inducible protein 10; KC, keratinocyte chemoattractant; LIF, leukemia inhibitory factor; MCP‐1, monocyte chemoattractant protein 1; ODE, organic dust extract; PLN, popliteal lymph node; TNF‐Tg, tumor necrosis factor transgenic.

## CONFLICTS OF INTEREST

The authors declare that there are no conflicts of interest.

## AUTHOR CONTRIBUTIONS

Li Xiong read a number of relevant literatures and wrote the manuscript as well as concluded the table. Liang Xiong revised grammar mistakes and made expression of language more native. Hong Ye ensured the language had its fluency and logic. Wan‐Li Ma did the final proofreading to get the manuscript more completeness.

## Data Availability

The authors confirm that the data supporting this review are available within the paper and on request from the corresponding author.
